# Fate and speciation of NO_x_ in an arid climatic region: factors assessment

**DOI:** 10.1007/s10661-025-14077-4

**Published:** 2025-05-08

**Authors:** Mohammad Abdullah Alolayan, Litty Mary Abraham, Ashraf Azmi Ramadan

**Affiliations:** 1 Department of Environmental Sciences, College of Life Sciences, Kuwait University, P. O. Box 5969, Safat, 13060 State of Kuwait; 2 Environmental Pollution and Climate Program, Environmental & Life Sciences Research Center, Kuwait Institute for Scientific Research, P. O. Box 24885, Safat, 13109 State of Kuwait

**Keywords:** NO_x_, NO_2_, NO, Oxidation, Fate of NO_x_, Modeling, Machine learning

## Abstract

NO and NO_2_ continuously recycle in the lower atmosphere through a complex series of reactions involving NO, VOCs, NO_2_, and O_3_. Therefore, the NO_2_/NO_x_ ratio can be utilized in dispersion models as an important substitute to understand the fate of NO and NO_2_ in the atmosphere. In this work, random forest regression was used to analyze the significance of meteorological parameters that affect the prediction of the NO_2_/NO_x_ in Kuwait’s three distinct regions—rural, urban, and industrial for the years 2004 to 2014, where different sources of pollution are present in each of these areas. The NO_2_/NO_x_ ratio did not change much over time for all the studied locations. The measured mean NO_2_ concentration in the urban regions is two times higher than that in the industrial area, implicating vehicular sources as the major contributor to air pollution compared to stationary sources. The coefficient of determination for NO_2_/NO_x_ for the four monitoring stations ranged between 0.62 and 0.81. Results indicate that although extreme temperature and intense solar radiation conditions are prominent in each of these areas, wind speed is the relatively important feature that significantly affects the NO_2_/NO_x_ ratio in urban areas.

## Introduction

Industrialization, along with the exploitation of natural resources as part of urbanization, has put a toll on the environment. The signs of these pollutions are more apparent with the deaths associated with ambient outdoor air pollution. According to the World Health Organization (WHO), the combined effects of ambient and household air pollution are associated with 6.7 million premature deaths annually (WHO, 2023). Air pollution causes a range of chronic and acute illnesses, including lung cancer, cardiovascular diseases, and chronic respiratory disorders (Bhat et al., 2021; Taghizadeh et al., 2023). Exposure to NO_2_ can deplete the tissue’s antioxidant defenses and might result in an injury or inflammation (Jarvis et al., 2010).

Pollutants that undergo a chemical change in the atmosphere cause complications. This is relevant if nitrogen dioxide (NO_2_) and nitric oxide (NO) are present (Ravina et al., 2022). Seven compounds make up the family of NO_x_ (Table 1) (Blaszczak, 1999). However, NO and NO_2_ are the dominant compounds. The presence of NO_x_ depends on the formation of the thermal NO as a precursor (Mollenhauer & Johnson, 2010). NO_x_ compounds are emitted mainly from combustion sources, with 90–95% in the NO form and 5–10% in the NO_2_ form (Jarvis et al., 2010). N_2_O easily undergoes oxidation in the presence of O_3_ and produces NO. Similarly, the volatile organic compounds (VOC) from power plants and vehicles oxidize NO to form NO_2_ and increase the NO_2_/NO_x_ ratio (Berezina, et al., 2020; Blaszczak, 1999). When a photon beam falls on these NO_2_ molecules during daytime, these NO_2_ create O_3_ molecules. Therefore, the presence of less O_3_ to titrate NO during early morning hours lowers the conversion of NO to NO_2_ (Kimbrough et al., 2017).

**Table 1:** Oxides of nitrogen

Formula	Compound name
NO	Nitric oxide
N_2_O_2_	Dinitrogen dioxide
N_2_O	Nitrous oxide
N_2_O_3_	Dinitrogen trioxide
NO_2_	Nitrogen dioxide
N_2_O_4_	Dinitrogen tetroxide
N_2_O_5_	Dinitrogen pentoxide

Automobiles and power plants account for 50% and 20% of the two most significant sources of anthropogenic NO_x_, respectively (Blaszczak, 1999). According to a study, the power plants in Kuwait were behind 105 Gg of NO_x_ emissions in 2016 (Ramdan, 2021). Other natural and anthropogenic sources include forest fires, grass fires, petroleum refineries, glass manufacture, and cement manufacture (Blaszczak, 1999). A study conducted in Saudi Arabia assessed the hourly and daily changes in concentrations of NO_x_, NO, and NO_2_ in the ambient air. The study explored the correlation between these changes and meteorological variables such as temperature, wind speed, relative humidity, and pressure. The results revealed that temperature and wind speed had negative correlation coefficients, while relative humidity had positive correlation coefficients with NO_x_, NO, and NO_2_ (Gasmi et al., 2017).

From 1995 to 2010, a rise in NO_2_/NO_x_ was observed for 61 European cities (Grange et al., 2017). A study showed that NO_2_/NO_x_ in Switzerland increased from 14% in 1992 to 23% in 2004 due to the increase in primary NO_2_ road traffic emissions (Hueglin et al., 2006). Another study found the oxidation of NO to NO_2_ to be more prevalent within the first 5 km from the plume and then declines further by a factor of 10–20 (Middleton et al., 2007). However, the highest reaction occurs at the edges of the plume and less at the core as there is no O_3_. A more recent study showed the conversion of NO to NO_2_ and the mixing of these emissions within the atmosphere to have a considerable impact on the NO_2_/NO_x_ ratio even at a distance of 20 m from the edge of the roadway (Kimbrough et al., 2017). In that study, the authors investigated the chemical progression of NO_x_ from power plants over 1000 km from the source. The authors concluded that the dispersion of NO_x_ was more on the land than over the sea. The authors emphasized the importance of looking more thoroughly at the availability of models that can accurately simulate specific species concentrations inside the plumes as dispersion modifies the plume’s concentration, which impacts the reaction rate (Kallend, 1995). The variables that influence the oxidation of NO_x_ of the plume are the time of emission, dispersion rate, and season. High temperature and wind speed also influence the NO conversion for the power station plumes (Luhana et al., 2007).According to a study, the NO_2_/NO_x_ ranges between 0.25 and 0.35 from a near-road monitor in Las Vegas (Bryant, et al., 2017). The following results were observed from the analysis of the NO_2_/NO_x_ in urban and rural regions of the United Kingdom (UK). The NO_2_/NO_x_ in rural parts of the UK was close to 0.85, while for urban areas, it was less, i.e., about 0.59. The NO_2_/NO_x_ is larger in rural locations due to the dispersion and more energetic interactions with the available O_3_, which result in higher NO_2_ levels. On the contrary, proximity to the source enhances the NO value and thus lowers NO_2_/NO_x_ ratios in urban areas (Bower et al., 1993).

NO quickly transforms to NO_2_ in the environment (Chaney et al., 2011). Therefore, it is not possible to model NO directly using dispersion models. However, modeling the NO_2_/NO_x_ can express the speciation between NO and NO_2_ (Kimbrough et al., 2017). Accordingly, the NO can be estimated based on the predicted NO_2_/NO_x_. This study examines the relative importance of factors that influence the NO_2_/NO_x_ in an arid region where extreme temperatures, elevated solar radiation, and dry air exist. This analysis was carried out in three different locations: urban, industrial, and rural areas where different emission sources are situated and at different distances.

## Materials and methods

The study was conducted in Kuwait, which is known to have an arid climate with the temperature during the summer months (June–August) exceeding 50 °C and the annual rainfall averaging 112 mm per year (Alolayan et al., 2013) (Table 2).

**Table 2:** Annual averages of meteorological parameters of Kuwait

Parameter	Fall	Winter	Spring	Summer
Temperature (°C)	27	15	27	38
Wind speed (mph)	6.8	7.3	7.5	8.8
Median wind direction* (degrees)	241	257	188	27
Wind gust (m/s)	5	5	5	6
Solar (w/m^2^)	216	263	311	382
Relative humidity (%)	36	59	35	21
Pressure (mbar)	1001	1006	999	987
Dew point* (°F)	47	41	43	43
Visibility* (statute mile)	6	6	5	5
Dust storm* (%)	13	7	20	32

^*^Alolayan et al. (2013)

In numerous studies, machine learning has been proven to be robust in modeling and forecasting air quality (Alkabbani et al., 2022; Liu et al., 2019; Shaw, et al., 2022). In this study, models were developed to predict NO_2_/NO_x_ using machine learning and the random forest regression method. Anaconda Spyder (version-5), a Python-based programming software, was used for the same. A previous study demonstrated that in the absence of emissions information, employing a correlated proxy with emissions in modeling can improve the prediction of pollutant’s ambient concentrations (Alolayan et al., 2023). Accordingly, weather parameters and the daily amount of fuel burned in power plants were included in the model as input variables.

**Fig. 1:**
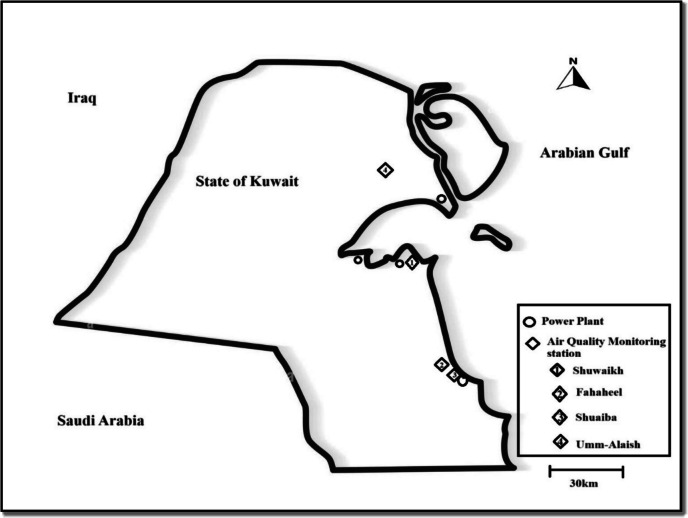
Locations of the four air quality monitoring stations

Meteorological data and concentrations of NO, NO_2_, and NO_x_ were collected from 4 monitoring stations in the State of Kuwait from 2004 to 2014 (Fig. 1). The monitoring stations belong to the Kuwait Environmental Public Authority (KEPA). Station 1 and Station 2 are located in urban areas, Al-Shuwaikh City (47.932437, 29.317731) and Al-Fahaheel City (48.116014, 29.080329), respectively. However, close to Station 2, there is a refinery located approximately 4 km away. Station 3 is in the Al-Shuaiba industrial city (48.153106, 29.038469) and adjacent to a large power plant. Station 4 is located in a rural area called Umm Al-Aish (47.669337, 29.690902) (Matar et al., 2020).

The meteorological data consists of measurements of temperature (°C), barometric pressure (mbar), wind speed (m/s), relative humidity (%), global solar radiation (W/m^2^), and wind direction (degree). The daily averages were used for all variables except for wind direction, where the median was used to represent the prevailing wind. Since data features with different scales might distort models and reduce their accuracy, the data were normalized between 0 and 1 for wind direction. Also, the wind direction was included in the modeling as a categorical variable with eight classes or vectors. Daily data on the quantity of each type of fuel—heavy fuel oil, crude oil, gas oil, and natural gas—burnt by each of the six power plants were retrieved from Kuwait’s Ministry of Electricity and Water (MEW). The data set was split into 75% for training the model and 25% for testing.

## Results and discussions

The average daily levels of NO and NO_2_ are elevated at stations 1 and 2 due to their urban settings, whereas Station 4, which is not near any sources, records the lowest daily levels of NO and NO_2_. The average daily ratio of NO_2_/NO_x_ concentration for the four monitoring sites from 2004 to 2014 exhibits a consistent trend (Table 3). The World Health Organization air quality guidelines for NO_2_ levels are 25 µg/m^3^ (13 ppb) on a daily basis and 10 µg/m^3^ (5 ppb) annually (Kan, 2022). The available daily concentrations of NO_2_ exceeded the relevant WHO standard for all the stations except for Station 4. A study showed that O_3_ concentration in Kuwait was within the national and international standards (Al-Hemoud, et al., 2021). This suggests that the amount of emitted NO_2_ is abundant and predominantly exceeds that resulting from the photochemical reaction between NO and O_3_. Looking at the mean concentration of NO and NO_2_ over the 10 years, one can notice that NO_2_ concentrations exceeded those of NO in all stations except for Station 4. Studies have shown that Kuwait’s high NO_2_ levels were attributed to vehicular emissions (mainly those from heavy-duty diesel vehicles), especially during peak hours (Al-Hemoud, et al., 2021; Shehab & Moinuddin, 2020). Furthermore, the consumption of natural gas is a substantial contributor to NO_2_ emissions (Al-Fadhli et al., 2019). A significant factor in the high level of NO_2_ in the area is the use of natural gas for power generation by the Shuaiba and Shuwaikh power plants.

**Table 3:**
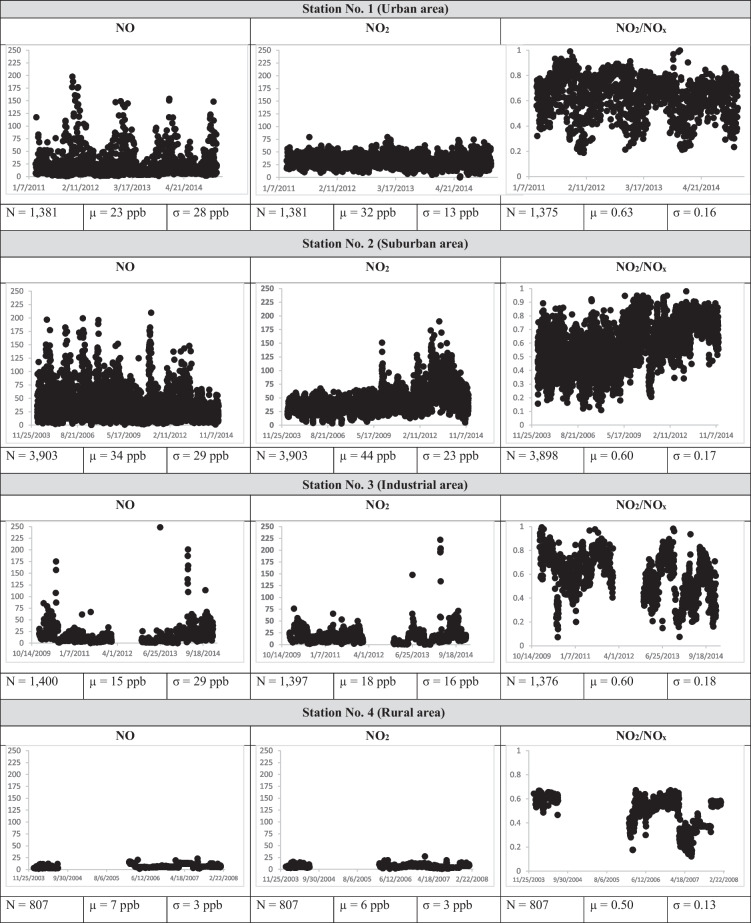
Daily levels of NO, NO_2_, and NO_2_/NO_x_ (*N* = sample size; *µ* = mean; *σ* = standard deviation)

**Fig. 2:**
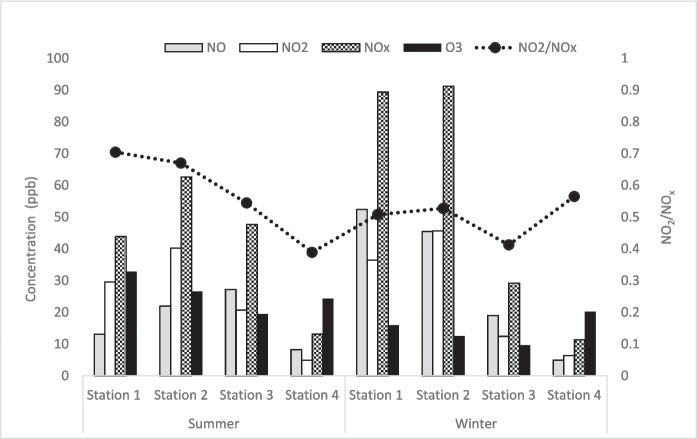
Seasonal variation in NO, NO_2_, NO_x_, O_3_, and NO_2_/NO_x_

The seasonal variation (summer and winter) in NO, NO_2_, NO_x_, and the ratio of NO_2_/NO_x_ over the four stations was studied and is depicted in the Fig. 2. NO_x_ exhibits a distinct seasonal pattern between the summer and winter periods. NO_x_ concentrations are significantly higher during the winter months compared to the summer months. During the summer months, higher O_3_ level is found due to the photochemical reaction involving strong solar radiation; however, this is less pronounced during the winter months. This finding aligns with a similar study conducted in New Jersey (Roberts-Semple et al., 2012). Moreover, the NO_2_/NO_x_ ratio is higher in the summer months, indicating a higher level of oxidation.

The coefficient of determination (*R*^2^) for NO_2_/NO_x_ for the four monitoring stations was between 0.62 and 0.81 (Table 4). The mean NO_2_/NO_x_ ratios were the same for all the stations except at Station 4, which is in a rural area and far from major sources. Naturally, wind speed has significant importance as higher wind speeds cause pollutants to be flushed out more quickly, while lower wind speeds allow more time for the reaction, hence higher NO_2_ (Kimbrough et al., 2013; Ravina et al., 2022). Wind speed is the most significant factor behind oxidation in Stations 1 and 2. For these stations, the average wind speed is 3.25 m/s (Station 1) and 1.86 m/s (Station 2). The percentage of the relative importance of wind speed is 45% and 21% at Stations 1 and 2, respectively (Table 4). However, this is less obvious for Stations 3 and 4. Also, considering Station 2 is in an urban area and close to distinct pollution sources, i.e., refinery, one can understand the higher NO_2_ concentrations (Gahalla et al., 2018).

**Table 4:** Relative important feature values of the regression models

		Station 1			Station 2			Station 3			Station 4		
		Urban			Urban			Industrial			Rural		
		NO_2_/NO_x_	NO_2_	NO	NO_2_/NO_x_	NO_2_	NO	NO_2_/NO_x_	NO_2_	NO	NO_2_/NO_x_	NO_2_	NO
*R*^2^		0.62	0.42	0.57	0.69	0.67	0.57	0.76	0.60	0.11	0.81	0.43	0.68
Sources (%)		44	40	35	69	73	44	74	71	66	76	56	66
Weather (%)	Temperature	3	3	6	4	3	5	12	5	16	6	5	9
	Pressure	-	-	-	-	-	-	-	-	-	5	2	9
	Solar	-	-	-	-	-	-	-	-	-	7	19	5
	R. humidity	4	5	4	4	3	5	5	16	4	2	3	4
	Wind speed	45	15	49	21	19	44	6	6	8	3	4	15
	Wind direction	4	37	6	2	2	3	3	3	5	1	1	3

In Station 3, which is situated adjacent to a power plant stack and along the coastline, the atmospheric temperature appears to be the important meteorological feature for predicting NO_2_/NO_x_. The elevated temperatures during the summer months result in higher energy usage due to the population’s heavy reliance on air conditioning systems. Higher energy demand means higher NO_2_ emissions from the power plants, which operate near maximum capacity during the summer and vice versa during the winter (Al-Hurban et al., 2021). Additionally, the relative humidity at Station 3 plays a significant role in predicting NO_2_ levels, given its coastal location. The high humidity and solubility of NO_2_ in comparison with NO lead to the formation of nitric acid (Tan & Piri, 2013).

Solar activity was found to be the feature with the highest importance for predicting the NO_2_/NO_x_ in Station 4. Solar, which derives the photochemical reaction, is stronger in this rural area. This is due to its location being in the desert and far (i.e., ~ 45 km from the nearest power plant) from any pollution sources. The lower background NO_2_ concentrations can be linked to the reduced NO_x_ emissions (Degraeuwe, et al., 2016). This is valid for Station 4, which is far from the direct impact of industrial emissions where the concentration of NO_2_ is lower than that of NO, indicating travel distance disperses the available NO_x_.

## Conclusion

The analysis of NO_2_/NO_x_ data from the four monitoring stations in Kuwait shows comparable ratios observed in other major cities of the world (Ravina et al., 2022). The NO_2_/NO_x_ showed a consistent trend over time for all the studied locations; this tendency can be attributed to the elevated NO_2_ levels. The 10-year average daily concentration of NO_2_ (44 ppb) at Station 2 confirmed that the concentration is 3.3 times higher than the WHO daily standard for NO_2_. The analysis indicates that urban and industrial locations, specifically Stations 1, 2, and 3, exhibited higher NO_2_/NO_x_ ratios than rural areas, such as Station 4. This phenomenon is brought on by higher NO_2_ emissions from local emission sources (refineries, power plants, and traffic). Our results indicate that although extreme temperatures and intense solar radiation are prevalent in each of the studied areas, considering the relative importance of weather parameters, wind speed is the significant feature for the dilution and photochemical reaction in the two major urban regions where Stations 1 and 2 are situated. Temperature is the relatively important feature for the industrial area where Station 3 is located (Station 3 is also adjacent to a power plant) and solar radiation in the rural area (Station 4). However, we believe that having emission inventory data would improve the accuracy of prediction and understanding of the fate of NO_x_. The findings from this study elaborate to the researchers in the field of air dispersion and modeling that temperature is not always the factor affecting the fate of NO_X_ regardless of the extreme temperature in arid areas.

In summary, this work found the importance of factors that influence the NO_2_/NO_x_ in the air of an arid region and the prevalence of excessive NO_2_ in urban and industrial regions.

## Data Availability

No datasets were generated or analysed during the current study.
